# Risk Factors and Mortality Outcomes in Elderly Patients With Bloodstream Infections: A Retrospective Analysis

**DOI:** 10.7759/cureus.65275

**Published:** 2024-07-24

**Authors:** Shahin Shah, Muhammad D Nadeem, Junaid Ali, Umair Ahmad, Abroo Mahmood, Zainab Ikhlas

**Affiliations:** 1 General Medicine, Medlife Medical Center, Abu Dhabi, ARE; 2 Medicine, Khyber Medical University, Peshawar, PAK; 3 General Medicine, Khyber Medical University, Peshawar, PAK; 4 Medicine, Khyber Pakhtunkhwa Health Department, Peshawar, PAK; 5 Internal Medicine, Advocare North Brunswick Medical Associates, North Brunswick, USA; 6 Internal Medicine, Faisal Hospital, Faisalabad, PAK

**Keywords:** bacteremia, mortality, utis, older adults, bloodstream infections

## Abstract

Background

The objective of our investigation was to evaluate the mortality rate and predictor factors that are associated with bloodstream infections (BSIs) in elderly patients who are admitted to the internal medicine ward.

Materials and methods

A retrospective cross-sectional analysis was conducted at a 550-bed tertiary care hospital in Peshawar, Pakistan, from January 2021 to June 2022. The study involved elderly inpatients aged 65 and older with positive culture results detected within two days of admission. Data collection involved demographic and patient-related risk variables, BSI-related risk factors, and environmental risk factors, with statistical analysis performed using the Statistical Package for the Social Sciences (IBM SPSS Statistics for Windows, IBM Corp., Version 26.0, Armonk, NY).

Results

Of the total study sample (n=186), 103 (55.4%) survived while 83 (44.6%) did not. The non-survivor group had a higher median Sequential Organ Failure Assessment (SOFA) score (6 vs. 2, p<0.0001) and Charlson Comorbidity Index (5 ± 2 vs. 3 ± 2, p<0.0001), with more frequent immunosuppression (25.3% vs. 8.7%, p=0.001). Additionally, gram-positive bacteria were more common in non-survivors (42% vs. 10%, p<0.0001), while gram-negative bacteria were more prevalent in survivors (73% vs. 36%, p=0.002).

Conclusions

Our research validates that BSI in older adults is a serious condition that is linked to a substantial death rate during hospitalization. The biggest determinant of death in older patients with BSI is the severity of clinical symptoms evaluated by the SOFA score upon admission. It is imperative to acknowledge that respiratory-induced BSIs are the most fatal, and patients who are hospitalized and admitted to the intensive care unit (ICU) are at an elevated risk.

## Introduction

Bacteremia is a severe condition that can be life-threatening, resulting in an estimated eight million deaths annually [1]. The elderly are at a higher risk, with mortality rates spanning from 14% to 37% [2]. This condition is the result of the invasion of harmful bacteria into the bloodstream, where they proliferate and produce toxic substances, resulting in a widespread inflammatory reaction throughout the body [3]. The imperative necessity for early detection and treatment to reduce mortality is underscored by the rapid progression and high fatality rate of bacteremia. The immune systems of elderly patients are particularly susceptible to bacteremia due to their diminished immune state, which hinders their ability to combat infections. Furthermore, the presence of subtle symptoms in older individuals can make it challenging to identify bacteremia, further complicating the clinical detection process. Their overall vulnerability, in conjunction with the distinctive manner in which it is presented, leads to increased mortality rates and less favourable outcomes [4]. Consequently, it is imperative to promptly identify and intervene in order to improve the outcomes of this vulnerable population.

During hospitalisation, elderly patients with bacteremia are at an elevated mortality risk, with comorbidities and advanced age being critical predictors of outcomes. According to research, the mortality rate is significantly increased by advanced age, which is a significant risk factor for systemic infections with drug-resistant organisms [5]. Bacteremia is associated with a poor prognosis and a higher long-term mortality in very elderly patients aged 80 years or later, particularly those over 90 years of age [6]. Comorbidities, including hypertension, diabetes mellitus, and chronic respiratory disease substantially influence the occurrence of nosocomial infections in elderly patients. The Charlson Comorbidity Index is a less accurate predictor of infection occurrence than the number of comorbidities [7]. These factors collectively underscore the significance of evaluating the susceptibility of geriatric patients to bacteremia and guiding treatment decisions to enhance outcomes by considering age and comorbidities.

Mortality rates are substantially determined by the severity of the initial illness, as evaluated by scoring systems such as Sequential Organ Failure Assessment (SOFA) and Acute Physiology and Chronic Health Evaluation (APACHE) [8,9]. The timely and precise administration of appropriate antibiotics is essential for enhancing patient outcomes [10]. Mortality rates are significantly influenced by the causative organism of bacteremia, particularly gram-negative bacteria, multi-drug resistant pathogens, and fungi [11,12]. In comparison to urinary tract infections (UTIs), infections that originate from sources such as the airways or gastrointestinal tract have a higher fatality rate. The outcomes of elderly individuals with bacteremia are significantly influenced by their nutritional status and functional status, as evidenced by factors such as albumin levels. It is imperative to assess the nutritional and functional status of patients to effectively inform therapeutic decisions. However, the incidence of bacteremia among elderly patients during hospitalization is frequently substantial, resulting in a substantial mortality rate. It is imperative to identify and resolve the indicators that indicate a patient's likelihood of mortality during their hospital stay to improve patient outcomes and reduce the burden on the healthcare system. This research will offer healthcare practitioners valuable insights that will enhance the treatment of bacteremia in this highly vulnerable group by providing a comprehensive understanding of these variables. The objective of our investigation was to evaluate the mortality rate and predictor factors that are associated with mortality in elderly patients who are admitted to the internal medicine ward.

## Materials and methods

A retrospective cross-sectional analysis was conducted at a 550-bed tertiary care hospital in Khyber Pakhtunkhwa (KPK), affiliated with Peshawar. The study sample comprised elderly inpatients, aged 65 years and above, who were admitted to the medicine inpatient department over the period from January 2021 to June 2022. The study sample was determined based on a global prevalence of bacteremia of 14% [2]. The calculated sample size was 186.

The laboratory reports from the microbiology department identified eligible individuals who had positive blood cultures within the first 48 hours of admission. Patients were excluded from the study if their blood cultures detected contaminants, if they had opted out, or if their medical records were incomplete. The study was approved by the Research and Ethical Committee of Amaan Medical Institute (reference: AMI-AAA-340), with a waiver granted for informed consent due to its retrospective character.

Clinically significant bacteremia was defined as the detection of non-commensal bacteria (bacteria that do not naturally exist within the host body and may cause disease or disruption when introduced in at least one blood sample). For patients with commensal bacteria, clinically significant bacteremia is determined by the presence of at least two positive culture findings on distinct dates during their hospital stay.

The data collection involved three distinct sections: section 1 encompassed demographic and patient-related risk variables, section 2 focused on bloodstream infection (BSI)-related risk factors, and section 3 addressed environmental risk factors.

Section 1 contains demographic information such as age and gender as well as health status factors such as immunosuppression and the SOFA score. The following conditions are indicative of immunosuppression: having human immunodeficiency virus (HIV) and a chronic kidney disease (CD4+) and cell count below 200/mm³, taking drugs that suppress the immune system for organ transplant or autoimmune disease, taking corticosteroids for more than three months at a dose of 7.5 mg of prednisolone or more, and having a low neutrophil count below 500/mm³. Further factors that could increase the risk included active solid or blood cancers, chronic kidney failure (defined as a glomerular filtration rate of less than 60 mL/min or the need for dialysis), severe dementia, and prolonged bed rest for more than three days while hospitalised.

In section 2, we included the following elements: healthcare-associated bloodstream infections (HCA-BSI) as defined by recent hospitalisation, dialysis, intravenous therapy, or residence in a long-term care facility; microorganism type (gram-positive, gram-negative, fungal, polymicrobial); infection source (urinary, respiratory, intra-abdominal, other, unknown), and the necessity of surgical or endoscopic intervention to control the infection's source.

Section 3 deals with recent hospital admissions, previous hospital admissions, and antibiotic therapy within the past 30 days. Patients were categorised into two groups: survivors, who were discharged alive, and non-survivors, who died during hospitalisation regardless of the cause and duration of their stay.

The mean ± standard deviation (SD) or median (interquartile range) was used to represent the descriptive statistics for continuous variables, while the number and percentage were used for categorical variables. To compare survivors and non-survivors, we used statistical tests that were specific to the variables we were studying. The Chi-squared or Fisher's exact tests were used to evaluate categorical variables, while the Student's t-test or Mann-Whitney U test were used to analyse continuous variables, according to their distribution. BSI mortality in the elderly was studied using regression analysis. In addition to p-values and 95% confidence intervals, the study also supplied odds ratios (ORs). A p-value < 0.05 was used as the operational definition of statistical significance. The Statistical Package for the Social Sciences (IBM SPSS Statistics for Windows, IBM Corp., Version 26.0, Armonk, NY) was used to perform the analysis.

## Results

The study sample (n=186) was divided into two groups: survivors (n=103) and non-survivors (n=83). The mean age was slightly higher in the non-survivor group (73 years, range 65-87) compared to the survivor group (71 years, range 68-84), but this difference was not statistically significant (p=0.214). The gender distribution was consistent across categories, with 85 males comprising 46% of the total sample: 35 (42%) of non-survivors and 50 (49%) of survivors (p=0.345).

The length of hospital stay was comparable between the groups; however, substantial disparities were observed in clinical scores and comorbidities. Non-survivors had a significantly higher SOFA score compared to survivors (p=0.00). The Charlson Comorbidity Index was also markedly higher in non-survivors (mean 5 ± 2) than in survivors (mean 3 ± 2, p=0.00). Immunosuppression was significantly more prevalent in the non-survivor group (25.3%) compared to the survivor group (8.7%) (p=0.001).

Non-survivors exhibited a higher prevalence of chronic renal insufficiency (60.2% vs. 36.9%, p=0.001) and dementia (31.3% vs. 16.5%, p=0.022). The functional status of the two groups was significantly different (p=0.00), with bedridden status being considerably more prevalent among non-survivors (60.2%) than survivors (9.7%). Additionally, the frequency of recent hospitalizations (30.1%) and ICU admissions (81.9%) was significantly higher in non-survivors (p=0.012 and p=0.01, respectively) (Table 1).

**Table 1:** Comparison of demographic and clinical characteristics of the study groups SOFA: Sequential Organ Failure Assessment; ICU: intensive care unit; * P< 0.05

Characteristics	Total (n=186)	Non-survivor Group (n=83)	Survivor Group (n=103)	p-value
Age (years) (mean and range)	72 (65-82)	73 (65-87)	71 (68-84)	0.21
Gender				0.35
Male	85 (46%)	35 (42%)	50 (49%)	
Females	101 (54%)	48 (58%)	53 (51%)	
Duration of hospitalization (days)	12 (7-24)	10 (5-15)	15 (8-25)	0.20
SOFA score	3 (2-4)	6 (4-8)	2 (1-3)	0.00*
Charlson Comorbidity Index	4 ± 2	5 ± 2	3 ± 2	0.00*
Immunosuppression condition	30 (16%)	21 (25.3%)	9 (8.73%)	0.00*
Cancer	69 (37%)	35 (42.2%)	34 (33%)	0.20
Chronic renal insufficiency	88 (47%)	50 (60.2%)	38 (36.9%)	0.00*
Dementia	43 (23%)	26 (31.3%)	17 (16.5%)	0.02
Bedridden status	60 (32%)	50 (60.2%)	10 (9.7%)	0.04*
ICU admission	92 (49.5%)	68 (81.92%)	24 (23.3%)	0.01*
Recent hospitalization (<1 month)	40 (22%)	25 (30.1%)	15 (14.6%)	0.01
Recent antibiotic use (<1 month)	35 (19%)	20 (24.1%)	15 (14.6%)	0.12

**Figure 1:**
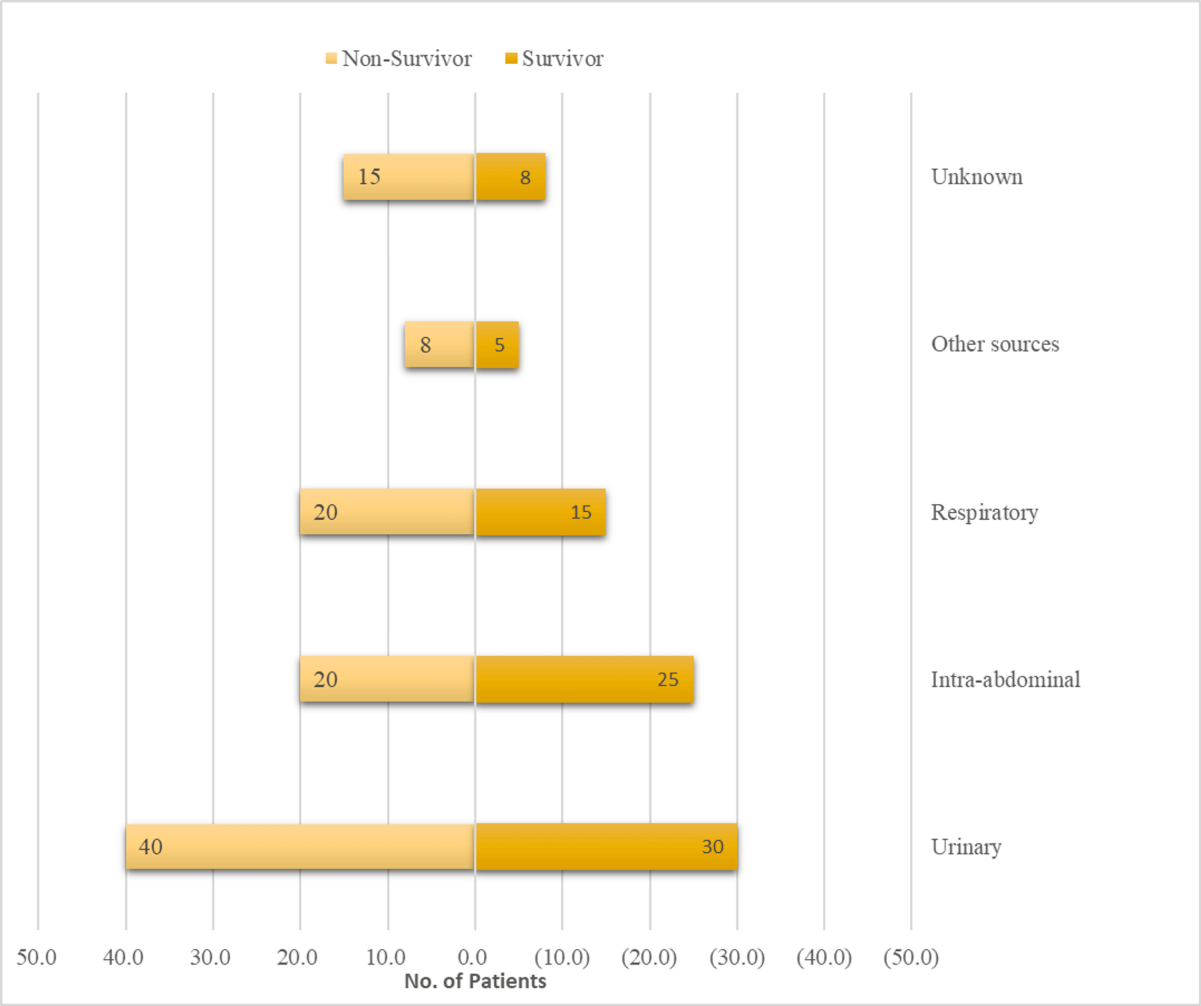
Comparison of sources of infection between groups

The source of infection varied significantly between the non-survivor group (n=83) and the survivor group (n=103). UTIs were the most common source in 70 cases (38%). Notably, they were more prevalent in the non-survivor group (48%) compared to the survivor group (29.1%), and this difference was statistically significant (p=0.012). Intra-abdominal infections were the next most common, with 45 cases (24%) overall. However, there were no significant differences in their distribution between the non-survivor and survivor groups (p=0.948). Respiratory infections were slightly more common in non-survivors (24%) than survivors (14.6%), but this difference wasn't significant (p=0.131). Infections from unknown sources were higher in non-survivors (18%) versus survivors (7.8%), a considerable difference (p=0.034). Other sources, like skin or foreign material, were less frequent but showed a non-significant trend towards more occurrences in non-survivors (10%) than survivors (4.9%) (p=0.157) (Figure 1).

**Figure 2:**
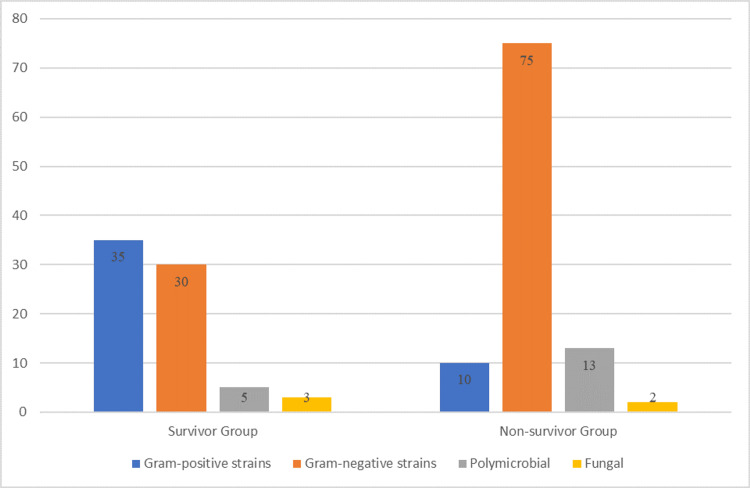
Comparison of bacterial distribution between groups

Significant differences in the prevalence of bacterial strains were observed between the non-survivor group (n=83) and the survivor group (n=103) as shown in Figure 2. Gram-positive strains were notably more common in the non-survivor group, identified in 35 patients (42%) compared to just 10 patients (10%) in the survivor group, a highly significant difference (p<0.0001). Conversely, gram-negative strains were more prevalent in the survivor group, with 75 patients (73%) affected, compared to 30 patients (36%) in the non-survivor group, also a significant difference (p=0.002).

Polymicrobial infections were found in 18 patients (10%) overall, with a higher frequency in the survivor group (13 patients, 13%) compared to the non-survivor group (five patients, 6%), showing a significant difference (p=0.027). Fungal infections were relatively rare and did not differ significantly between the groups, with three cases (4%) in the non-survivor group and two cases (2%) in the survivor group (p=0.64).

The regression analysis, as shown in Table 2, identified several critical factors associated with mortality in elderly patients with BSI. Age showed a borderline significant link with mortality (p=0.05), suggesting a slight risk increase with age. Gender had no significant impact on mortality, with females having similar odds to males (p=0.97). Hospitalization duration also did not significantly affect mortality risk (p=0.28). Important predictors of mortality included higher SOFA scores (p=0.00) and Charlson Comorbidity Index scores (p=0.00). Bedridden status was linked to a much higher risk of mortality (p=0.00). Chronic renal insufficiency showed a non-significant trend towards higher mortality risk (p=0.06).

**Table 2:** The regression analysis of demographic, clinical and environmental factors associated with mortality among the study sample SOFA: Sequential Organ Failure Assessment; ICU: intensive care unit; UTIs: urinary tract infections; * P<0.05

Variables	OR (95% Confidence Interval)	p-value
Age	1.06 (0.99-1.12)	0.05
Gender		
Males		
Females	1.01 (0.54-1.90)	0.97
Length of hospitalization (days)	0.99 (0.1-1.01)	0.28
SOFA score	0.70 (0.42-2.11)	0.00*
Charlson Comorbidity Index	1.24 (1.1-1.92)	0.00*
Immunosuppression	0.17 (0.30-1.59)	0.59
Cancer	0.3 (0.49-1.78)	0.82
Chronic renal insufficiency	1.0 (0.96-3.39)	0.06
Dementia	1.36 (0.68-2.73)	0.38
Bedridden status	3.82 (2.03-7.20)	0.00*
Recent hospitalization (<1 month)	1.07 (1.02-2.19)	0.00*
ICU admission	1.34 (1.12-.58)	0.04*
Recent antibiotic use (<1 month)	0.75 (0.32-1.79)	0.52
Adequate empirical antibiotics	0.89 (0.46-1.70)	0.72
Infection Sources		
UTIs	1	
Respiratory	7.11 (2.94-17.20)	0.00*
Intra-abdominal	1.34 (0.50-3.61)	0.56
Other	2.83 (1.05-7.59)	0.04*
Pathogen Type		
Gram-positive	1	
Gram-negative	0.53 (0.27-1.04)	0.08
Polymicrobial	0.54 (0.16-1.86)	0.33
Fungal	2.64 (0.60-11.59)	0.20

Recent ICU admission was significantly associated with increased mortality risk (p=0.04), indicating the potential benefits of ICU care. Recent hospitalization (p=0.00) was also associated with mortality risk; however, recent antibiotic use (p=0.52) and adequate empirical antibiotics (p=0.72) did not significantly predict mortality. Infection source played a significant role, with respiratory infections linked to higher mortality (p<0.001), while intra-abdominal infections did not (p=0.56). Other infection sources also increased mortality risk (p=0.04).

Regarding pathogen type, gram-negative infections showed a trend towards lower mortality (OR 0.53, 95% CI 0.27-1.04, p=0.08), though not statistically significant. Polymicrobial (p=0.33) and fungal infections (p=0.20) also did not significantly affect mortality.

## Discussion

In this study, we examined mortality and its predictors among elderly patients with BSI admitted to a tertiary care hospital. Among 183 patients, significant clinical differences were observed between the non-survivor group (n=83) and the survivor group (n=103). BSI originated from respiratory infections were more common among non-survivors (24% vs. 14.6%, p=0.131), and infections from unknown sources were significantly higher in this group (18% vs. 7.8%, p=0.034). Gram-positive bacterial strains were significantly more prevalent in non-survivors (42% vs. 10%, p<0.0001), whereas gram-negative strains were more common in survivors (73% vs. 36%, p=0.002).

The results of our study are consistent with other research that suggests older adults are at a higher risk of dying from infections compared to younger groups [13,14]. Research has shown a significant correlation between the immunological condition of patients and their clinical results [15,16]. More precisely, several phases of immunosuppression are associated with diverse prognostic variables, including age, comorbidities, ICU hospitalization, infection sources, aetiology, antibiotic usage, presence of resistant organisms, underlying diseases, hypoalbuminemia, and shock. Research has repeatedly shown that age is a substantial risk factor for death caused by bacteremia [17-20]. Nevertheless, there is a lack of research on the factors that can predict death in older persons with weakened immune systems who have BSI.

ICU death rates are much higher in patients with BSIs [21]. There is an elevated risk of fatal infections and complications in patients admitted to ICUs, particularly those whose immune systems are already weak [22]. There is strong evidence that older diabetic patients with a history of ICU admissions are at increased risk of mortality due to bacterial infections, with age being a major determinant in this risk [23]. When patients with BSIs have hematopoietic stem cell transplantation, being hospitalized in the ICU greatly increases their risk of mortality [18,22]. We found that older patients with compromised immune systems are more likely to die within 90 days after admission to the ICU (p=0.00), lending credence to earlier findings that this is a distinct predictive risk factor.

Moreover, the excessive utilization of fluoroquinolones, broad-spectrum cephalosporins, and carbapenems is linked to a heightened probability of acquiring bacterial infections that are resistant to treatment, resulting in unfavourable consequences [24]. Although tigecycline is commonly used to treat BSIs, there are concerns about its effectiveness. A meta-analysis demonstrated that tigecycline provided only marginal advantages over traditional antimicrobial treatments for severe infections, exhibiting lower rates of treatment success compared to control medicines [25]. Furthermore, the use of tigecycline was found to be a substantial indicator of mortality within 28 and 90 days [24,26]. The investigation yielded consistent findings, establishing a strong correlation between recent antibiotic usage and mortality rates in older individuals.

The results of our research showed that individuals who did not survive had considerably higher SOFA scores (p=0.00), Charlson Comorbidity Index scores (p=0.00), and a higher percentage of them were bedridden (60.2% vs. 9.7%, p=0.00). The results of this study are consistent with prior research that has also found comparable associations between these clinical factors and mortality rates in older patients with BSI [27-29].

Non-survivors with higher SOFA scores exhibit more severe organ dysfunction, which has consistently been associated with worse outcomes in critically sick patients [27]. The Charlson Comorbidity Index, a metric that assesses the load of comorbid illnesses, has shown a substantial increase in those who did not survive, underscoring the influence of numerous chronic health problems on the probability of mortality [28]. Furthermore, the significant occurrence of being bedridden among individuals who did not survive highlights the importance of functional status as a crucial factor in determining survival. Individuals with restricted mobility are more prone to developing issues such as pressure sores and infections, which can worsen their already delicate state of health [7].

The results of our study showed that non-survivors had a higher occurrence of respiratory infections compared to survivors (24% vs. 14.6%). However, this difference did not reach statistical significance (p=0.131). The incidence of infections of undetermined origin was notably greater in the group of those who did not survive (18% vs. 7.8%, p=0.034). These observations indicate that the seriousness and source of infections have a critical impact on patient outcomes. Prior studies have demonstrated that respiratory infections and unknown sources of infection are linked to increased mortality rates due to their intricate nature and the difficulties they provide in terms of efficient care [28,30].

The groups also exhibited distinct variations in the microbiological profile of illnesses. Non-survivors had a substantially greater occurrence of gram-positive bacterial strains (42% vs. 10%, p<0.0001), but survivors were more prone to having gram-negative strains (73% vs. 36%, p=0.002). This is consistent with previous research that suggests gram-positive infections, namely those caused by methicillin-resistant *Staphylococcus aureus* (MRSA), have a greater death risk in elderly individuals. On the other hand, gram-negative infections, albeit severe, tend to have more favourable results since there are more efficient antibiotic treatments specifically designed for these types of bacteria [7]. The elevated mortality associated with gram-positive bacteria, particularly MRSA, underscores the necessity for timely and appropriate antibiotic treatment. Our study highlights the importance of considering multiple clinical, functional, and microbiological parameters when evaluating the prognosis of older patients with BSI. The results emphasize the necessity for comprehensive care methods that not only focus on treating the immediate illness but also consider the underlying comorbidities and functional level of these susceptible patients. One weakness of our study is its retrospective form, which could result in selection bias and restrict the capacity to prove causality. Furthermore, the use of a single-centre setting may limit the applicability of our findings to different healthcare settings.

## Conclusions

Our research validates that BSI in older adults is a serious condition that is linked to a substantial death rate during hospitalization. The biggest determinant of death in older patients with BSI is the severity of clinical symptoms evaluated by the SOFA score upon admission. It is imperative to acknowledge that respiratory-induced BSIs are the most fatal, and patients who are hospitalized and admitted to the ICU are at an elevated risk. On the other hand, UTIs are the most frequent cause of BSI, but they pose a relatively lower level of danger. Further research should prioritize the development of precise therapies aimed at enhancing outcomes in this population at high risk.
